# Biomarkers of systemic disease burden and outcomes after transcatheter tricuspid edge-to-edge repair

**DOI:** 10.1186/s12872-026-06392-6

**Published:** 2026-08-03

**Authors:** Johannes Schlegl, Marwin Bannehr, Michael Lichtenauer, Tanja Kücken, Alexander Krutz, Vera Paar, Michael Neuß, Anja Haase-Fielitz, Christian Butter, Christoph Edlinger

**Affiliations:** 1 Department of Cardiology, Heart Center Brandenburg, Brandenburg Medical School (MHB) Theodor Fontane, Ladeburger Straße 17, Bernau bei Berlin, 16321 Germany; 2 Faculty of Health Sciences (FGW), Joint Faculty of the University of Potsdam, the Brandenburg Medical School Theodor Fontane and the Brandenburg Technical University Cottbus-Senftenberg, Cottbus, Germany; 3 Clinic of Internal Medicine II, Department of Cardiology, Paracelsus Medical University of Salzburg, Salzburg, Austria; 4 Institute of Social Medicine and Health System Research, Otto von Guericke University Magdeburg, Magdeburg, Germany

**Keywords:** Tricuspid regurgitation, Transcatheter edge-to-edge repair, Right-sided heart failure, GDF-15, suPAR

## Abstract

**Background:**

Risk stratification after transcatheter tricuspid edge-to-edge repair (T-TEER) remains challenging, particularly in patients with advanced right-sided heart failure and systemic disease burden. Biomarkers reflecting inflammation, stress response and multiorgan dysfunction may provide additional prognostic information in this setting.

**Methods:**

This prospective single-centre cohort study included 84 consecutive patients undergoing T-TEER for severe tricuspid regurgitation using the TriClip or PASCAL system. Baseline concentrations of growth differentiation factor-15 (GDF-15), soluble urokinase plasminogen activator receptor (suPAR), and NT-proBNP were measured prior to intervention. The primary endpoint was all-cause mortality at 12 months. Secondary endpoints included cardiovascular rehospitalisation and a combined cardiovascular endpoint. Prognostic performance was assessed using receiver operating characteristic and tertile-based Kaplan-Meier analyses. Owing to the limited number of events, all analyses were considered exploratory.

**Results:**

During 12-month follow-up, all-cause mortality occurred in 10 patients (11.9%), while cardiovascular rehospitalisation was observed in 29 patients (34.5%). GDF-15 demonstrated good discriminative performance for all-cause mortality (AUC 0.823, 95% CI 0.714–0.932, *p* = 0.001), whereas suPAR showed moderate prognostic discrimination (AUC 0.742, 95% CI 0.605–0.878, *p* = 0.013). In contrast, NT-proBNP showed no significant discrimination for all-cause mortality (AUC 0.535, 95% CI 0.362–0.709, *p* = 0.720). Higher tertiles of both GDF-15 and suPAR were associated with reduced overall and rehospitalisation-free survival.

**Conclusion:**

Baseline GDF-15 and suPAR were associated with adverse outcomes after T-TEER and demonstrated greater prognostic discrimination than NT-proBNP in this exploratory cohort. These exploratory findings support further investigation of systemic stress and inflammation biomarkers for risk stratification in patients with severe tricuspid regurgitation.

**Supplementary Information:**

The online version contains supplementary material available at 10.1186/s12872-026-06392-6.

## Introduction

Severe tricuspid regurgitation (TR) is increasingly recognised as a clinically relevant valvular disorder associated with substantial morbidity and excess mortality, particularly in patients with advanced heart failure and right ventricular dysfunction [1, 2]. Functional TR is now recognised as an independent contributor to adverse clinical outcomes and impaired survival [2, 3]. In advanced disease, systemic venous congestion and right ventricular remodelling may contribute to multiorgan dysfunction [1, 4].

Because isolated tricuspid valve surgery is often not feasible in elderly and comorbid patients, transcatheter tricuspid interventions have emerged as less invasive treatment options for patients deemed inoperable or at high surgical risk [5, 6]. In particular, transcatheter tricuspid edge-to-edge repair (T-TEER) has demonstrated procedural feasibility and has been associated with reduction of TR severity, symptom improvement, and improved quality of life in clinical studies and real-world cohorts [5, 7]. Despite these advances, clinical outcomes after T-TEER remain heterogeneous, and reliable preprocedural risk stratification remains challenging [8].

Risk stratification in severe TR is complex because prognosis is influenced not only by valvular severity, but also by right ventricular dysfunction, systemic congestion, and multiorgan involvement. In addition, MELD-XI-based approaches integrating hepatorenal dysfunction with invasive haemodynamic parameters may further improve prognostic assessment after transcatheter tricuspid valve intervention [9]. Conventional biomarkers such as natriuretic peptides are well established in heart failure, but their prognostic performance may be limited in patients with predominant right-sided heart failure, concomitant renal dysfunction, and systemic venous congestion, which are common in advanced TR populations [10, 11].

Consequently, increasing interest has focused on biomarkers reflecting systemic stress, inflammation, and multiorgan dysfunction that may better capture the complex pathophysiology of advanced TR. Since advanced TR is characterised by chronic venous congestion, progressive hepatorenal dysfunction and systemic inflammatory activation, biomarkers reflecting these pathophysiological processes may provide prognostic information beyond conventional markers of myocardial wall stress. Growth differentiation factor-15 (GDF-15) is a stress-responsive cytokine associated with inflammation, oxidative stress, and tissue injury and has been linked to adverse outcomes in heart failure populations [12–14]. Similarly, soluble urokinase-type plasminogen activator receptor (suPAR), a marker of chronic immune activation and systemic disease burden, has been associated with adverse outcomes in patients with heart failure [15, 16].

Recent studies suggest that biomarker-based approaches may refine risk stratification in patients undergoing transcatheter tricuspid interventions, particularly when integrated with haemodynamic assessment [17–19]. In addition, contemporary registry and trial data have further highlighted the clinical relevance of patient selection and risk assessment in T-TEER populations [20–22]. However, most available studies have evaluated heterogeneous transcatheter tricuspid intervention populations or have focused on biomarker assessment in combination with invasive haemodynamic parameters. Whether biomarkers reflecting systemic disease burden retain their prognostic value in the more procedurally homogeneous population undergoing T-TEER, independent of the procedural heterogeneity of broader transcatheter tricuspid intervention cohorts, remains incompletely understood.

We therefore aimed to evaluate the association of circulating biomarkers reflecting systemic disease burden with clinical outcomes in a homogeneous cohort of patients undergoing T-TEER, with particular focus on GDF-15 and suPAR.

## Methods

### Study design and patient population

This prospective, single-centre observational cohort study included consecutive patients undergoing transcatheter tricuspid edge-to-edge repair (T-TEER) for symptomatic severe tricuspid regurgitation at a tertiary care heart centre. Patients were enrolled consecutively between 1 November 2022 and 30 November 2024. Severe tricuspid regurgitation was defined according to current echocardiographic recommendations. Patients were evaluated by an interdisciplinary heart team consisting of interventional cardiologists, cardiac surgeons, imaging specialists and anaesthesiologists. Patients were considered eligible for transcatheter tricuspid intervention if they had symptomatic severe tricuspid regurgitation despite guideline-directed medical therapy and were deemed at high surgical risk or inoperable.

Anatomical suitability for T-TEER was determined by comprehensive transthoracic and transoesophageal echocardiography according to contemporary guideline recommendations and manufacturer-specific selection criteria, including assessment of leaflet morphology, coaptation gap, leaflet tethering, jet location and imaging quality to determine procedural feasibility.

Inclusion criteria were age ≥ 18 years, symptomatic severe tricuspid regurgitation and suitability for transcatheter edge-to-edge repair based on anatomical assessment by transoesophageal echocardiography. Exclusion criteria included active infection, advanced malignancy with limited life expectancy, untreated significant left-sided valvular disease requiring primary intervention, or inability to provide written informed consent.

**Fig. 1:**
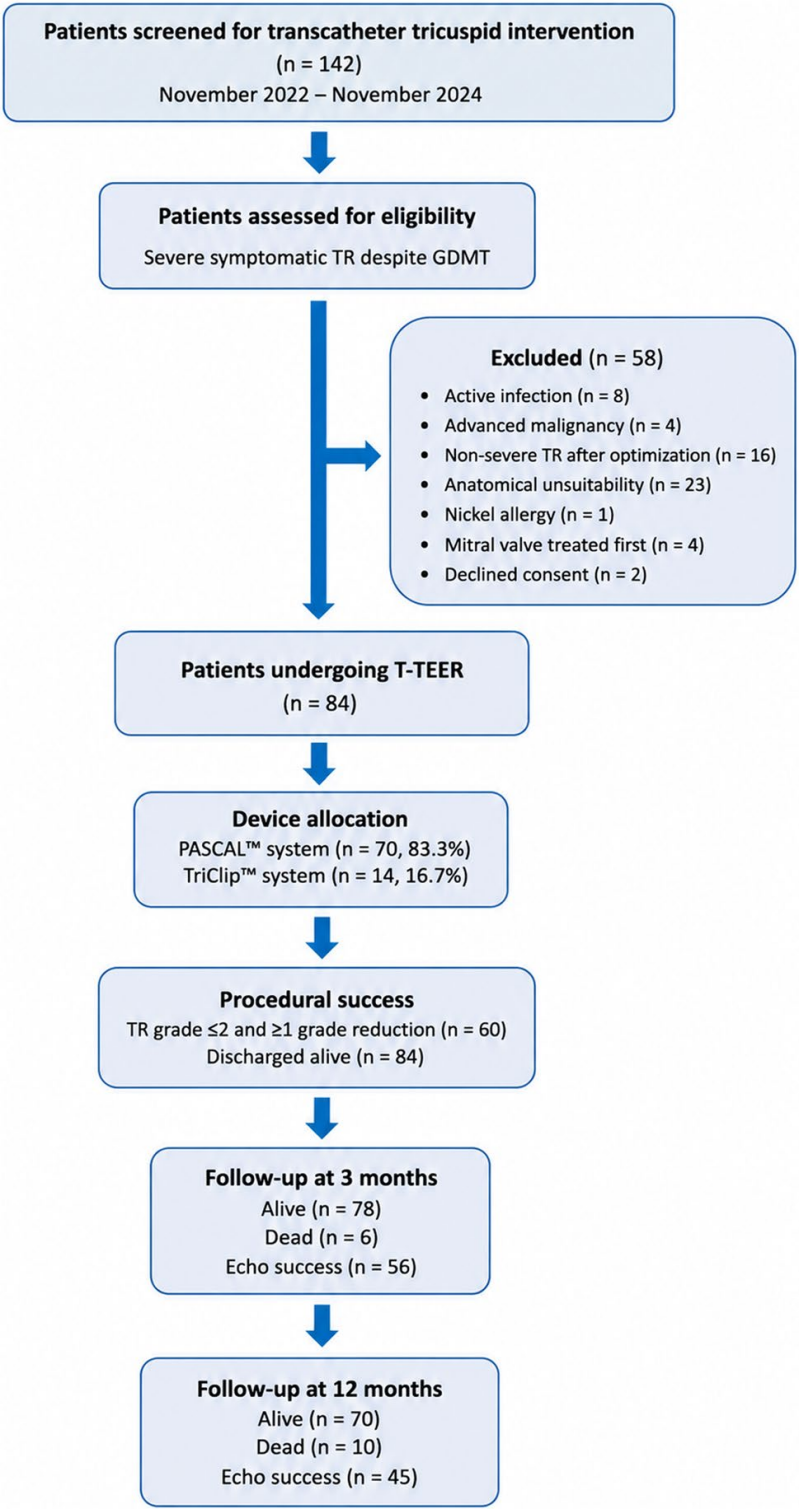
Flow chart of the study. Flow diagram showing patient screening, inclusion, intervention type, and follow-up to 12 months. Abbreviations: T-TEER = transcatheter edge-to-edge repair

A total of 84 consecutive patients undergoing transcatheter tricuspid edge-to-edge repair were included in the present analysis, as illustrated in the study flow chart (Fig. 1). As the present study was restricted to patients undergoing T-TEER, it represents a procedurally homogeneous cohort without inclusion of alternative transcatheter tricuspid valve interventions.

The study was conducted in accordance with the Declaration of Helsinki and approved by the institutional ethics committee (approval number E-01-20190503). All patients provided written informed consent prior to inclusion.

### Interventional procedure

All procedures were performed in a dedicated hybrid catheterisation laboratory under general anaesthesia with fluoroscopic and transoesophageal echocardiographic guidance. Vascular access was obtained via the femoral vein. Intraprocedural anticoagulation was achieved using unfractionated heparin according to institutional standards.

Transcatheter tricuspid edge-to-edge repair (T-TEER) was performed using either the TriClip ^TM^ system (Abbott Vascular, Santa Clara, CA, USA) or the PASCAL ^TM^ system (Edwards Lifesciences, Irvine, CA, USA), depending on anatomical suitability, leaflet morphology and operator discretion.

Both systems have been previously described and demonstrated procedural feasibility, reduction of tricuspid regurgitation severity and improvement in clinical outcomes in patients with severe tricuspid regurgitation in clinical trials and real-world registries [1–3].

Procedural success was defined as successful device implantation with at least a one-grade reduction in tricuspid regurgitation severity at discharge and a residual tricuspid regurgitation grade ≤ 2, assessed by transthoracic echocardiography according to current recommendations. Periprocedural complications were recorded during the index hospitalisation.

All echocardiographic examinations were performed by experienced cardiologists at a tertiary university-affiliated heart centre. Although no independent core laboratory was available, all operators had obtained the highest national level of echocardiographic certification and had received dedicated manufacturer-certified training in anatomical screening, patient selection, and contraindications for transcatheter tricuspid edge-to-edge repair.

### Blood sampling and biomarker analysis

Venous blood samples were obtained immediately prior to the interventional procedure following standardised venipuncture. Samples were centrifuged within 30 min and serum aliquots were stored at − 80 °C until analysis.

GDF-15 concentrations were quantified using the Human GDF-15 Quantikine ELISA Kit (Catalog No. DGD150; R&D Systems, Minneapolis, MN, USA), and suPAR concentrations were determined using the Human uPAR DuoSet ELISA (Catalog No. DY807; R&D Systems, Minneapolis, MN, USA), according to the manufacturers’ instructions. Laboratory personnel performing biomarker measurements were blinded to clinical data and outcomes.

NT-proBNP levels were determined using routine clinical immunoassays at the institutional laboratory. All measurements were performed in duplicate. Biomarker concentrations were analysed as continuous variables throughout all statistical analyses.

### Endpoints and follow-up

The primary endpoint of the study was all-cause mortality at 12 months after transcatheter tricuspid edge-to-edge repair.

Secondary endpoints included:

- cardiovascular rehospitalisation within 12 months after T-TEER,
- a combined cardiovascular endpoint consisting of cardiovascular rehospitalisation and all-cause mortality within 12 months after T-TEER.

Follow-up data were obtained from hospital records, outpatient visits and structured telephone interviews. Follow-up was performed at 3 and 12 months after the procedure.

### Statistical analysis

Continuous variables are presented as mean ± standard deviation or median with interquartile range, as appropriate. Categorical variables are presented as counts and percentages.

Receiver operating characteristic (ROC) curve analysis was performed to evaluate the prognostic discrimination of baseline biomarker levels including NT-proBNP for mortality and cardiovascular outcomes. The area under the curve (AUC) was calculated with corresponding 95% confidence intervals.

Patients were stratified according to tertiles of baseline GDF-15 and suPAR concentrations for Kaplan-Meier survival analysis. Differences between groups were compared using the log-rank test.

Spearman rank correlation analyses were performed to evaluate associations between GDF-15 and suPAR concentrations and clinically relevant baseline characteristics, including age, estimated glomerular filtration rate (eGFR), NT-proBNP, MELD-XI score and tricuspid annular plane systolic excursion (TAPSE).

Given the limited number of mortality events, extensive multivariable modelling was intentionally avoided to reduce the risk of model overfitting and unstable effect estimates. Accordingly, all analyses should be regarded as exploratory and hypothesis-generating.

A two-sided p-value < 0.05 was considered statistically significant. Statistical analyses were performed using SPSS Version 29 (IBM Corp., Armonk, NY, USA).

## Results

### Patient characteristics

A total of 84 consecutive patients undergoing transcatheter tricuspid edge-to-edge repair were included in the present analysis. Baseline demographic, clinical and echocardiographic characteristics are summarised in Table 1. Our study population consisted of elderly patients with advanced tricuspid regurgitation undergoing transcatheter intervention and represented a high-risk cohort with pronounced right-sided heart failure, substantial comorbidity burden and frequent multiorgan involvement, particularly impaired renal function. Left ventricular systolic function was largely preserved or only mildly reduced, whereas right ventricular function was commonly impaired, consistent with advanced right-sided volume overload and disease progression. Overall, the cohort reflects a severely affected tricuspid regurgitation population with limited functional capacity, high systemic disease burden and advanced right heart dysfunction. Baseline concentrations of GDF-15 and suPAR are summarised in Table 1.

**Table 1:** Baseline characteristics

Variable	Value
Demographics	*n* = 84
Sex, male [%]	46.4
Age [years], median [IQR]	82.0 (IQR 78.0–85.0)
Body mass index [kg/m²], median [IQR]	25.9 (IQR 24.1–28.7)
**Intervention**	
TriClip [%]	16.7
PASCAL [%]	83.3
**Scores**	
TRI-Score, median [IQR]	5 (IQR 4–6)
MELD-XI-Score, median [IQR]	13 (IQR 11–16)
EuroSCORE II, median [IQR]	4.5 (IQR 2.6–6.9)
**Echocardiographic parameters**	
LVEF [%], mean ± SD	50.6 ± 10.1
TAPSE [mm], median [IQR]	16.0 (IQR 14.0–18.0)
TR grade, median [IQR]	4 (IQR 3–5)
**Haemodynamic measurements**	
PVR [WU], mean ± SD	2.3 ± 1.0
Cardiac output [L/min], mean ± SD	4.3 ± 1.2
**Comorbidities**	
Arterial hypertension [%]	70.2
Type 2 diabetes mellitus [%]	66.7
Coronary artery disease [%]	58.3
COPD [%]	21.4
eGFR [mL/min/1.73 m²], median [IQR]	41.0 (IQR 32.0–48.0)
Concomitant valvular disease [%]	47.5
Of which treated [%]	64.3
**Functional status**	
6-minute walking distance [m], median [IQR]	151 (IQR 0–333)
6-minute walking distance < 100 m [%]	36.6
**Biomarkers stratified by outcome**	
**GDF-15 [pg/mL]**,** median [IQR]**	
Survivors (*n* = 74)	1534.1 (IQR 1106.9–2299.8)
Non-survivors (*n* = 10)	2781.4 (IQR 1995.9–3595.4)
No CV rehospitalisation (*n* = 55)	1473.6 (IQR 1051.8–2060.2)
CV rehospitalisation (*n* = 29)	1930.3 (IQR 1671.2–2942.6)
**suPAR [pg/mL]**,** median [IQR]**	
Survivors (*n* = 74)	3789.5 (IQR 2804.4–4491.7)
Non-survivors (*n* = 10)	4604.8 (IQR 4125.5–5274.4)
No CV rehospitalisation (*n* = 55)	3504.8 (IQR 2741.5–4385.6)
CV rehospitalisation (*n* = 29)	4186.7 (IQR 3611.3–4718.8)

Baseline demographic and clinical characteristics of all patients. Continuous variables are shown as mean ± SD or median (IQR), as appropriate; categorical variables are presented as n (%)

*Abbreviations*: *eGFR *estimated glomerular filtration rate, *MELD XI* Model for End-Stage Liver Disease Excluding INR, *CKD* chronic kidney disease, *COPD* chronic obstructive pulmonary disease, *TR* tricuspid regurgitation, *T-TEER* tricuspid valve transcatheter edge-to-edge repair, *TAPSE* tricuspid annular plane systolic excursion

### Clinical outcomes

During 12-month follow-up, all-cause mortality occurred in 10 of 84 patients (11.9%), while cardiovascular rehospitalisation due to decompensated heart failure was documented in 29 of 84 patients (34.5%). The combined endpoint of all-cause mortality or cardiovascular rehospitalisation occurred in 31 patients (36.9%). Procedural success was achieved in 60 patients (71.4%), whereas 24 patients (28.6%) did not meet the predefined echocardiographic success criteria at discharge. Clinical events were descriptively more frequent among patients without procedural success (all-cause mortality: 20.8% vs. 8.3%; cardiovascular rehospitalisation: 50.0% vs. 28.3%, respectively).

**Fig. 2:**
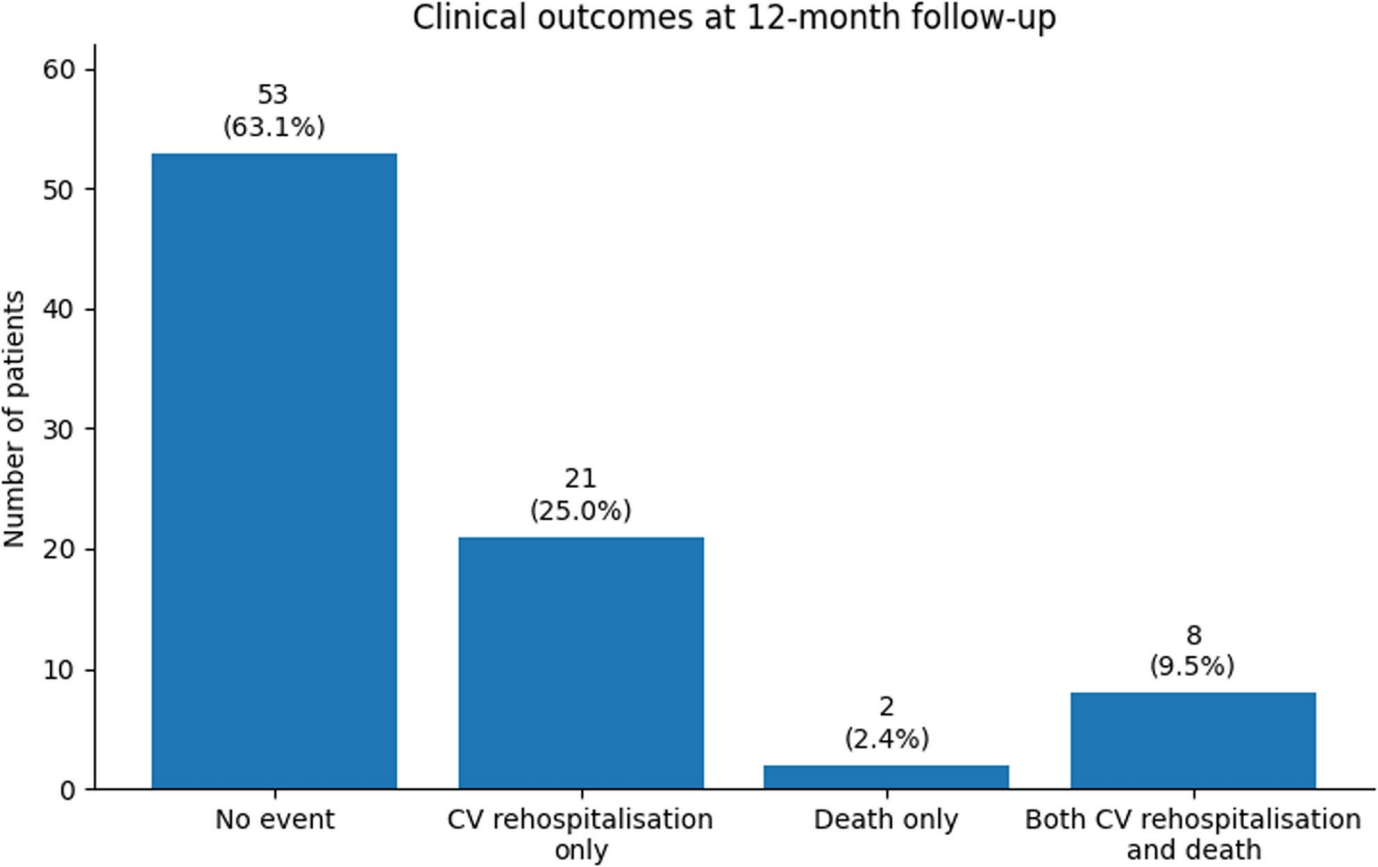
Clinical outcomes at 12-month follow-up. Bars represent the number and percentage of patients without clinical events, with cardiovascular rehospitalisation only, all-cause mortality only, and both cardiovascular rehospitalisation and all-cause mortality during 12-month follow-up

Of these, 21 patients experienced cardiovascular rehospitalisation only, two patients died without prior cardiovascular rehospitalisation, and eight patients experienced both cardiovascular rehospitalisation and death during follow-up (Fig. 2). Among patients with rehospitalisation, the median time to first event was 3 months (IQR 2–7).

### ROC analysis

Receiver operating characteristic (ROC) curve analysis was performed to assess the discriminative performance of baseline biomarker levels for mortality and cardiovascular outcomes at 12 months.

**Fig. 3:**
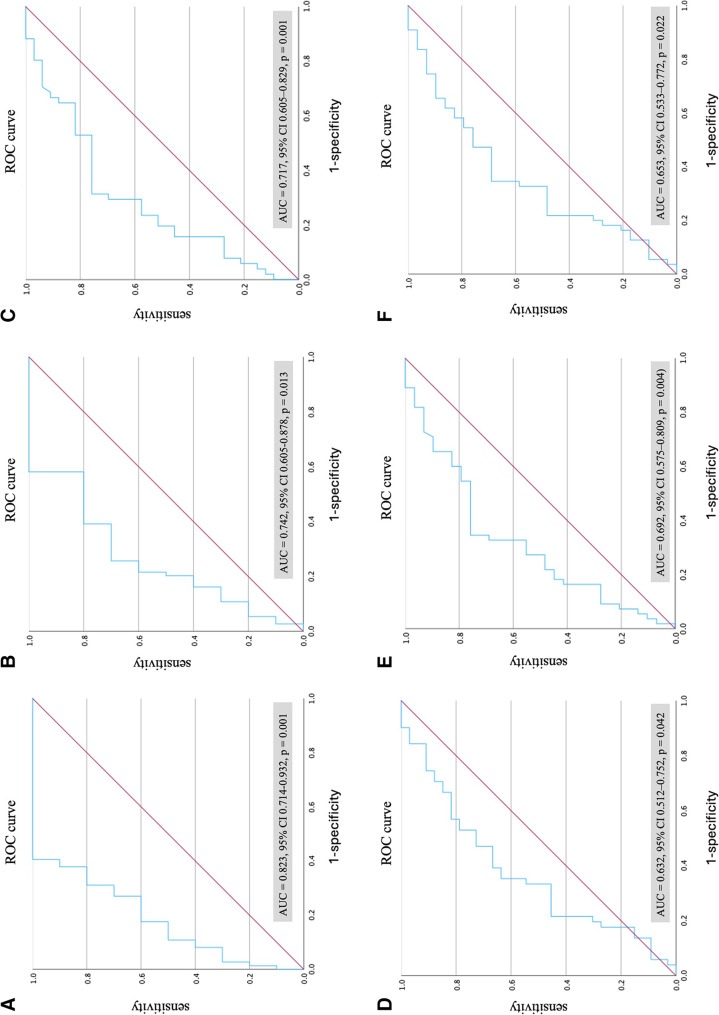
**a** ROC analysis of GDF-15 for all-cause mortality at 12 months. Receiver operating characteristic curve illustrating the prognostic performance of GDF-15. Abbreviations: ROC = receiver operating characteristic; AUC = area under the curve; CI = confidence interval; GDF-15 = growth differentiation factor-15. **b** ROC analysis of suPAR for all-cause mortality at 12 months. Receiver operating characteristic curve illustrating the prognostic performance of suPAR. Abbreviations: ROC = receiver operating characteristic; AUC = area under the curve; CI = confidence interval; suPAR = soluble urokinase plasminogen activator receptor. **c** ROC analysis of GDF-15 for the combined cardiovascular endpoint at 12 months. **d** ROC analysis of suPAR for the combined cardiovascular endpoint at 12 months. **e** ROC analysis of GDF-15 for cardiovascular rehospitalization at 12 months. **f** ROC analysis of suPAR for cardiovascular rehospitalization at 12 months

For the prediction of all-cause mortality, GDF-15 demonstrated good discriminative ability (AUC = 0.823, 95% CI 0.714–0.932, *p* = 0.001), whereas suPAR showed moderate discriminative performance (AUC = 0.742, 95% CI 0.605–0.878, *p* = 0.013; Fig. 3a and b). In contrast, NT-proBNP showed no significant prognostic discrimination for all-cause mortality (AUC = 0.535, 95% CI 0.362–0.709, *p* = 0.720).

For prediction of the combined cardiovascular endpoint defined as cardiovascular rehospitalisation or all-cause mortality at 12 months, GDF-15 showed moderate discriminative ability (AUC = 0.717, 95% CI 0.605–0.829, *p* = 0.001), while suPAR demonstrated lower discriminative performance (AUC = 0.632, 95% CI 0.512–0.752, *p* = 0.042; Fig. 3c and d). NT-proBNP likewise showed no significant prognostic discrimination for the combined cardiovascular endpoint (AUC = 0.578, 95% CI 0.448–0.708, *p* = 0.228).

For the prediction of cardiovascular rehospitalisation due to decompensated heart failure at 12 months, GDF-15 demonstrated modest discriminative performance (AUC = 0.692, 95% CI 0.575–0.809, *p* = 0.004), whereas suPAR showed limited discriminative ability (AUC = 0.653, 95% CI 0.533–0.772, *p* = 0.022; Fig. 3e and f). Similarly, NT-proBNP did not significantly discriminate cardiovascular rehospitalisation (AUC = 0.545, 95% CI 0.405–0.686, *p* = 0.495). Patients were stratified according to tertiles of baseline GDF-15 and suPAR concentrations.

### Kaplan-Meier survival analysis

Kaplan-Meier survival analysis was performed after stratification of patients according to tertiles of baseline GDF-15 and suPAR concentrations.

**Fig. 4:**
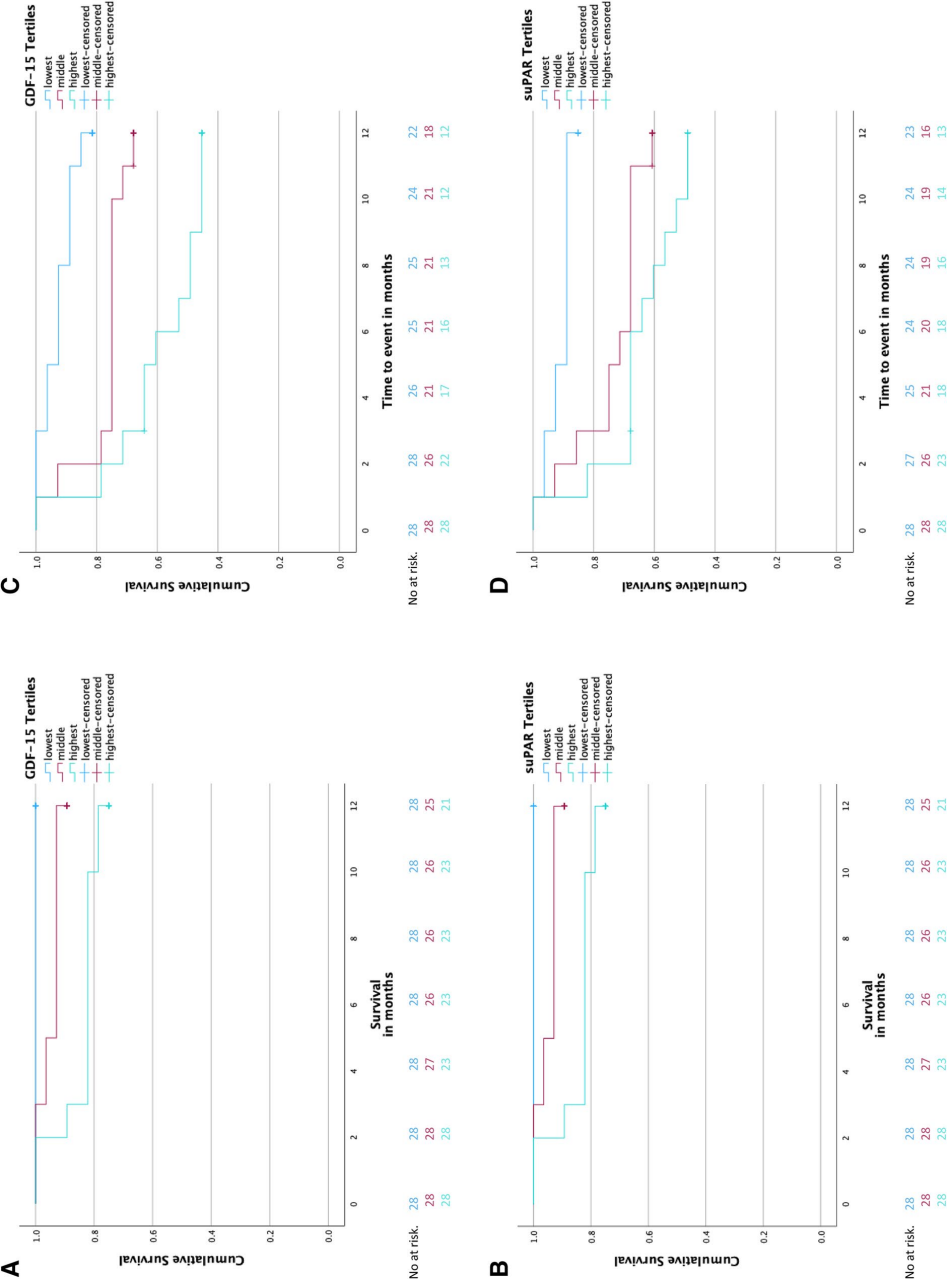
**a** Kaplan–Meier survival curves for all-cause mortality during 12-month follow-up according to tertiles of baseline GDF-15 concentrations. Survival curves for 12 months all-cause mortality according to GDF-15 tertiles. Differences between tertiles were assessed using the log-rank test (Mantel-Cox: χ² = 9.203, df=2, *p* = 0.010). Abbreviations: GDF-15 = growth differentiation factor-15. **b** Kaplan–Meier survival curves for all-cause mortality during 12-month follow-up according to tertiles of baseline suPAR concentrations. Survival curves for 12 months all-cause mortality according to suPAR tertiles. Differences between tertiles were assessed using the log-rank test (Mantel-Cox: χ² = 8.620, df=2, *p* = 0.013). Abbreviations: suPAR = soluble urokinase plasminogen activator receptor. **c** Kaplan–Meier curves for cardiovascular rehospitalisation-free survival during 12-month follow-up according to tertiles of baseline GDF-15 concentrations. Kaplan-Meier curves illustrating rehospitalisation-free survival according to GDF-15 tertiles. Differences between tertiles were assessed using the log-rank test (Mantel-Cox: χ² = 9.620, df=2, *p* = 0.008). Abbreviations: GDF-15 = growth differentiation factor-15. **d** Kaplan–Meier curves for cardiovascular rehospitalisation-free survival during 12-month follow-up according to tertiles of baseline suPAR concentrations. Kaplan-Meier curves illustrating rehospitalisation-free survival according to suPAR tertiles. Differences between tertiles were assessed using the log-rank test (Mantel-Cox: χ² = 7.129, df=2, *p* = 0.028). Abbreviations: suPAR = soluble urokinase plasminogen activator receptor

Patients in higher tertiles of GDF-15 showed significantly reduced survival compared with those in lower tertiles. Similarly, higher suPAR tertiles were associated with worse survival. When stratifying patients according to tertiles, GDF-15 tertiles showed significant differences in overall survival (log-rank χ² = 9.203, *p* = 0.010), with comparable findings observed for suPAR tertiles (log-rank χ² = 8.620, *p* = 0.013; Fig. 4a and b).

Consistently, GDF-15 tertiles showed significant differences in rehospitalisation-free survival (log-rank χ² = 9.620, *p* = 0.008), with similar results observed for suPAR tertiles (log-rank χ² = 7.129, *p* = 0.028; Fig. 4c and d).

### Correlation analyses

Spearman rank correlation analyses were performed to assess the relationship between circulating biomarker concentrations and clinically relevant baseline characteristics (Supplementary Table 1). GDF-15 demonstrated moderate correlations with impaired renal function (eGFR: *r* = − 0.548, *p* < 0.001), NT-proBNP (*r* = 0.419, *p* < 0.001), and MELD-XI score (*r* = 0.435, *p* < 0.001), whereas no significant associations were observed with age or TAPSE. Similarly, suPAR correlated with eGFR (*r* = − 0.380, *p* < 0.001), NT-proBNP (*r* = 0.265, *p* = 0.015), and MELD-XI score (*r* = 0.326, *p* = 0.003), while no significant correlations with age or TAPSE were found.

### Summary of biomarker performance

Overall, GDF-15 demonstrated the strongest and most consistent prognostic performance across all investigated clinical endpoints, whereas suPAR showed moderate discriminative ability. In contrast, NT-proBNP did not significantly discriminate all-cause mortality, cardiovascular rehospitalisation, or the combined cardiovascular endpoint in the present T-TEER cohort.

## Discussion

In this prospective cohort of patients undergoing transcatheter tricuspid edge-to-edge repair, biomarkers reflecting systemic stress and inflammation, particularly GDF-15 and suPAR, were associated with adverse clinical outcomes during follow-up. In contrast, baseline NT-proBNP did not demonstrate significant prognostic discrimination for mortality, cardiovascular rehospitalisation, or the combined cardiovascular endpoint in the present cohort. These findings support the concept that, in severe tricuspid regurgitation, biomarkers reflecting systemic disease burden may complement conventional biomarkers for risk stratification.

Severe tricuspid regurgitation is increasingly recognised as a progressive disease associated with right ventricular dysfunction, systemic venous congestion and multiorgan involvement, ultimately resulting in impaired functional capacity, recurrent hospitalisation and increased mortality [4–6]. Beyond valvular insufficiency itself, advanced TR is characterised by chronic congestion affecting the liver, kidneys and systemic circulation, as well as inflammatory and endothelial alterations [5, 7, 8]. This broader systemic disease profile may help explain why outcomes after transcatheter tricuspid interventions remain heterogeneous despite successful reduction of TR severity.

Although T-TEER has consistently been shown to improve symptoms and quality of life, prognosis appears to remain strongly influenced by the underlying stage of right-sided heart failure and systemic organ involvement at the time of intervention [9–11]. Risk stratification in this setting is therefore challenging, because prognosis is determined not only by valvular severity but also by the extent of right ventricular dysfunction, haemodynamic impairment and multiorgan dysfunction. Natriuretic peptides such as NT-proBNP are well-established biomarkers in heart failure and reflect myocardial wall stress and volume overload [12, 13]. However, in patients with advanced TR and predominant right-sided heart failure, their prognostic value may be attenuated by renal dysfunction, systemic congestion and the complex haemodynamic profile of right heart failure [14–16]. Consistent with this concept, NT-proBNP showed no significant prognostic discrimination across the investigated clinical endpoints in the present cohort.

By contrast, GDF-15 and suPAR reflect broader biological pathways including inflammation, oxidative stress, endothelial dysfunction and systemic disease burden [17–19]. Chronic venous congestion in advanced TR contributes to progressive hepatorenal dysfunction and systemic inflammatory activation, processes that may be captured more comprehensively by these biomarkers than by natriuretic peptides alone. Elevated baseline levels of GDF-15 and suPAR may therefore reflect more advanced systemic disease despite technically successful valve intervention. In this context, the prognostic value of these biomarkers may reflect cumulative extracardiac and cardio-renal-hepatic disease burden rather than isolated cardiac impairment. This interpretation is further supported by the observed correlations of both biomarkers with renal function, MELD-XI score and NT-proBNP, suggesting that GDF-15 and suPAR reflect systemic disease severity rather than isolated cardiac dysfunction.

Our findings are consistent with previous studies demonstrating prognostic value of next-generation biomarkers in heart failure and transcatheter valve interventions [20–23]. They also align with recent data suggesting that integration of circulating biomarkers with invasive haemodynamic parameters may further improve risk stratification in transcatheter tricuspid interventions [24, 25].

In contrast to previous reports that evaluated heterogeneous transcatheter tricuspid intervention populations or integrated biomarker assessment with invasive haemodynamic variables, the present study focused exclusively on patients undergoing T-TEER. Restricting the analysis to this procedurally homogeneous treatment strategy allowed evaluation of biomarker performance in a clinically well-defined population eligible for edge-to-edge repair. The observation that GDF-15 and suPAR retained prognostic value in this selected cohort supports the robustness of these biomarkers across different transcatheter treatment settings.

From a clinical perspective, improved identification of patients at increased risk before intervention remains highly relevant for patient selection, procedural timing and postprocedural management. Biomarker-based risk stratification may complement clinical, echocardiographic and haemodynamic assessment and may help identify patients who require closer follow-up and intensified heart failure management after intervention. Although patients without procedural success experienced descriptively higher event rates, the limited number of outcome events precluded meaningful subgroup analyses. At the same time, elevated biomarker levels should not be interpreted as markers of procedural futility, but rather as indicators of advanced systemic disease and higher overall risk.

In summary, in patients undergoing transcatheter tricuspid edge-to-edge repair, biomarkers reflecting systemic stress and inflammation, particularly GDF-15 and suPAR, were associated with adverse clinical outcomes. The present findings should be considered exploratory and hypothesis-generating. They suggest that biomarkers reflecting systemic disease burden may complement established clinical, echocardiographic and cardiac biomarkers for risk stratification in patients undergoing T-TEER.

### Limitations

This study has several limitations. First, this was a single-centre observational study with a relatively small sample size, which may limit the generalisability of the findings. Second, the relatively low number of clinical events limited statistical power. To reduce the risk of model overfitting and unstable effect estimates, extensive multivariable analyses were intentionally not performed. Accordingly, the present analyses were restricted to univariable statistical approaches and should be considered exploratory and hypothesis-generating.

Third, biomarker measurements were performed only at baseline prior to the intervention, and serial biomarker measurements during follow-up were not available. Serial measurements might provide additional information regarding dynamic changes after tricuspid valve repair and their potential prognostic relevance.

Fourth, although descriptive comparisons according to procedural success were performed, the limited number of outcome events precluded meaningful subgroup analyses and formal statistical comparisons.

Fifth, echocardiographic follow-up was incomplete at 12 months, which may have introduced attrition bias for imaging-based analyses, although clinical outcome assessment was complete for all patients.

Sixth, the predominance of the PASCAL system compared with the TriClip system precluded meaningful device-specific analyses and may have limited the generalisability of device-related findings.

Finally, although clinically relevant variables were considered, residual confounding due to unmeasured variables cannot be excluded.

## Conclusion

In patients undergoing transcatheter tricuspid edge-to-edge repair, biomarkers reflecting systemic stress and inflammation, particularly GDF-15 and suPAR, were associated with adverse clinical outcomes during 12-month follow-up after T-TEER. In contrast, baseline NT-proBNP did not demonstrate significant prognostic discrimination in the present cohort. Although the findings should be considered exploratory and hypothesis-generating, they suggest that biomarkers reflecting systemic disease burden may complement established clinical, echocardiographic and cardiac biomarkers for risk stratification in selected patients undergoing T-TEER. Further studies in larger, multicentre cohorts are warranted to validate these findings and to further define the potential role of systemic biomarkers in future risk stratification strategies for patients undergoing T-TEER.

## Supplementary Information

Supplementary Material 1.

## Data Availability

No datasets were generated or analysed during the current study.

## Abbreviations

GDF-15: Growth differentiation factor-15
suPAR: Soluble urokinase plasminogen activator receptor
TR: Tricuspid regurgitation
T-TEER: Transcatheter tricuspid edge-to-edge repair
ROC: Receiver operating characteristic
AUC: Area under the curve
CI: Confidence interval
HF: Heart failure
LVEF: Left ventricular ejection fraction
TAPSE: Tricuspid annular plane systolic excursion
PVR: Pulmonary vascular resistance
eGFR: Estimated glomerular filtration rate
MELD-XI: Model for End-Stage Liver Disease excluding INR
