# Supplementary material for: Biomarkers of systemic disease burden and outcomes after transcatheter tricuspid edge-to-edge repair
Source: BMC Cardiovasc Disord. 2026 Aug 3;26:653. doi: 10.1186/s12872-026-06392-6 (PMC13435473; doi:10.1186/s12872-026-06392-6)
Supplement: Supplementary file 1 [file 12872_2026_6392_MOESM1_ESM.docx]

**Supplementary Table 1. Spearman rank correlation analyses between circulating biomarkers and clinically relevant baseline characteristics**

| **Variable** | **GDF-15, ρ** | **P value** | **suPAR, ρ** | **P value** |
| --- | --- | --- | --- | --- |
| Age | 0.049 | 0.657 | −0.017 | 0.881 |
| eGFR | −0.548 | <0.001 | −0.380 | <0.001 |
| NT-proBNP | 0.419 | <0.001 | 0.265 | 0.015 |
| MELD-XI score | 0.435 | <0.001 | 0.326 | 0.003 |
| TAPSE | −0.126 | 0.268 | −0.080 | 0.484 |

Values are presented as Spearman's rank correlation coefficient (ρ).

**Abbreviations:** eGFR, estimated glomerular filtration rate; GDF-15, growth differentiation factor-15; MELD-XI, Model for End-stage Liver Disease excluding INR; NT-proBNP, N-terminal pro-B-type natriuretic peptide; suPAR, soluble urokinase plasminogen activator receptor; TAPSE, tricuspid annular plane systolic excursion.
